# ZeroReg3D: a zero-shot registration pipeline for 3D consecutive histopathology image reconstruction

**DOI:** 10.1117/1.JMI.12.4.044002

**Published:** 2025-08-05

**Authors:** Juming Xiong, Ruining Deng, Jialin Yue, Siqi Lu, Junlin Guo, Marilyn Lionts, Tianyuan Yao, Can Cui, Junchao Zhu, Chongyu Qu, Yuechen Yang, Mengmeng Yin, Haichun Yang, Yuankai Huo

**Affiliations:** a Vanderbilt University, Department of Electrical and Computer Engineering, Nashville, Tennessee, United States; b Vanderbilt University, Department of Computer Science, Nashville, Tennessee, United States; c Weill Cornell Medicine, New York, United States; d Vanderbilt University, Department of Pathology, Microbiology and Immunology, Nashville, Tennessee, United States

**Keywords:** image registration, histology, pathology, deep learning, 3D reconstruction

## Abstract

**Purpose:**

Histological analysis plays a crucial role in understanding tissue structure and pathology. Although recent advancements in registration methods have improved 2D histological analysis, they often struggle to preserve critical 3D spatial relationships, limiting their utility in both clinical and research applications. Specifically, constructing accurate 3D models from 2D slices remains challenging due to tissue deformation, sectioning artifacts, variability in imaging techniques, and inconsistent illumination. Deep learning-based registration methods have demonstrated improved performance but suffer from limited generalizability and require large-scale training data. In contrast, non-deep-learning approaches offer better generalizability but often compromise on accuracy.

**Approach:**

We introduce ZeroReg3D, a zero-shot registration pipeline that integrates zero-shot deep learning-based keypoint matching and non-deep-learning registration techniques to effectively mitigate deformation and sectioning artifacts without requiring extensive training data.

**Results:**

Comprehensive evaluations demonstrate that our pairwise 2D image registration method improves registration accuracy by ∼10% over baseline methods, outperforming existing strategies in both accuracy and robustness. High-fidelity 3D reconstructions further validate the effectiveness of our approach, establishing ZeroReg3D as a reliable framework for precise 3D reconstruction from consecutive 2D histological images.

**Conclusions:**

We introduced ZeroReg3D, a zero-shot registration pipeline tailored for accurate 3D reconstruction from serial histological sections. By combining zero-shot deep learning-based keypoint matching with optimization-based affine and non-rigid registration techniques, ZeroReg3D effectively addresses critical challenges such as tissue deformation, sectioning artifacts, staining variability, and inconsistent illumination without requiring retraining or fine-tuning.

## 1 Introduction

**Fig. 1:**
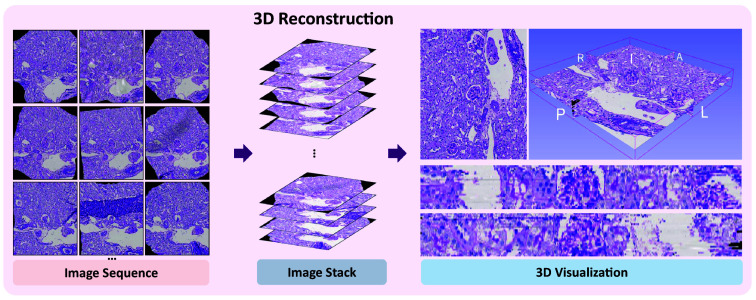
Overview. This figure shows a reconstructed 3D volume after alignment. The image sequence was stacked and subjected to 3D visualization to provide a comprehensive view.

Histology plays an essential role in both clinical diagnosis and biomedical research. Scientists and clinicians alike analyze tissue slides to identify key cellular structures and pathological features. In recent years, an increasing number of deep learning-based methods have been proposed to aid in tissue analysis and disease diagnosis.^1^^–^^4^ However, these methods, which rely on two-dimensional (2D) slices, fail to capture the intricate, three-dimensional (3D) spatial architecture of tissues present in histological samples, leading to a loss of information surrounding disease mechanisms^5^^,^^6^ and potential therapeutic targets.^7^ By visualizing the intricate spatial architecture of tissues in three dimensions, researchers and clinicians can gain deeper insights into disease mechanisms, progression, and potential therapeutic targets. However, the transition from 2D histological slices to comprehensive 3D models remains a significant challenge across multiple domains of pathology as shown in Fig. 1.

Although significant progress has been made toward accurate 3D reconstruction of 2D consecutive histopathology images,^8^ numerous obstacles persist in the field, including tissue deformation, sectioning artifacts, variability in staining techniques, and inconsistent illumination across sections.^9^ Tissue deformation arises from biological variability and the mechanical stresses involved in slicing, leading to misalignment between consecutive sections.^10^ Sectioning artifacts, such as tears or folds, further complicate the registration process, whereas heterogeneous staining and differing illumination conditions can obscure anatomical features necessary for accurate registration.^11^

In this paper, we introduce ZeroReg3D, a hybrid registration pipeline that integrates zero-shot deep learning and non-deep-learning registration methods for 3D reconstruction using routinely collected 2D WSIs. The proposed pipeline does not require retraining or finetuning by employing zero-shot XFeat^12^ key point extraction and matching, as well as affine registration and B-spline based deformable registration. Our 3D reconstruction method has been evaluated by mice whole kidney sections and human needle biopsy sections, demonstrating that our method outperforms existing registration strategies in both accuracy and robustness. The contributions of this paper are threefold:

• Holistic zero-shot deep learning and non-deep-learning design: Our method does not require additional retraining or finetuning on new data. The zero-shot deep learning based keypoint matching ensures broad applicability and facilitates easy integration across diverse histological datasets.
• Comprehensive studies: Our 3D reconstruction method has been evaluated by mice whole kidney sections and human needle biopsy sections, covering the two prevalent biopsy formats and different spices.
• Open-source deployment: The entire pipeline has been released as an open-source package, which is publicly available at https://github.com/hrlblab/ZeroReg3D.

## 2 Related Work

### 2.1 Deep Learning-based Registration

Deep-learning based methods leverage neural networks to learn robust feature representations that facilitate image registration by directly predicting correspondences between images. For instance, methods such as SuperGlue^13^ and OmniGlue^14^ utilize graph neural network architectures to refine feature matching by considering the contextual relationships among detected keypoints, resulting in more reliable global registration even under challenging conditions. Transformer-based approaches such as LoFTR^15^ establish dense, context-aware correspondences across image pairs without relying on explicit keypoint detection, whereas CNN-based frameworks such as XFeat^12^ offer efficient feature extraction particularly suited for resource-limited scenarios. Importantly, although these deep learning methods vary in architecture, they typically focus on estimating a single global transformation (e.g., a homography) for image registration, as demonstrated by DeTone et al.^16^

### 2.2 Non-deep Learning Based Registration

Non-deep learning-based methods form the traditional foundation of image registration by relying on handcrafted features and well-established mathematical models. These techniques include intensity-based approaches that utilize similarity metrics such as mutual information, cross-correlation, and structural similarity^17^^–^^19^ to determine the optimal registration between images. In addition, feature-based methods employing algorithms such as SIFT and ORB^20^^,^^21^ detect and match distinctive keypoints to compute transformation parameters for global registration. When it comes to capturing local, non-rigid deformations, methods such as B-splines,^22^ the Demons algorithm,^23^ and Thin Plate Splines (TPS)^24^ are employed to model spatially varying transformations. These approaches are often integrated into comprehensive software suites such as Advanced Normalization Tools (ANTs).^25^

### 2.3 Registration Methods in Pathology

In pathology, specialized registration methods have emerged to address unique challenges associated with serial histological section registration, including tissue deformation, missing sections, and staining variability. Map3D^26^ employs a registration-based framework integrating deep learning for the automated identification and association of large-scale 3D glomerular structures across serial renal histology sections, employing a quality-aware strategy to overcome these challenges. Similarly, DeeperHistReg^27^ combines deep learning feature extraction with traditional registration techniques to robustly align consecutive histopathology slides stained differently, facilitating precise registration critical for downstream pathological analyses.

## 3 Method

**Fig. 2:**
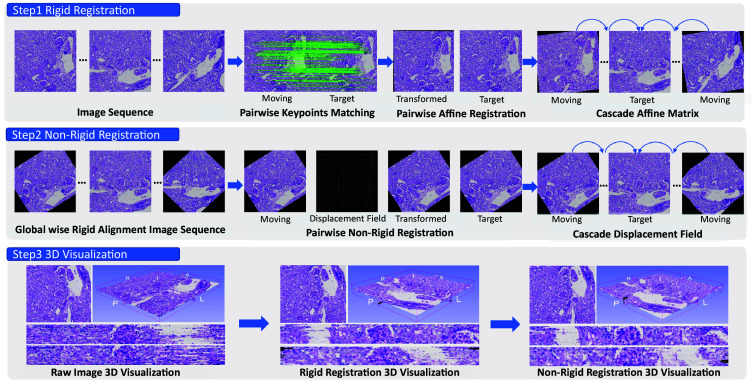
Method. This figure shows the entire pipeline and the visualization difference between raw image sequence, rigid registration image sequence, and non-rigid registration image sequence.

The entire framework of the proposed 3D registration is presented in Fig. 2. This pipeline consists of three sections: (1) keypoints extraction and matching, (2) affine registration, and (3) B-spline based non-rigid registration.

### 3.1 Keypoints Extraction and Matching

**Fig. 3:**
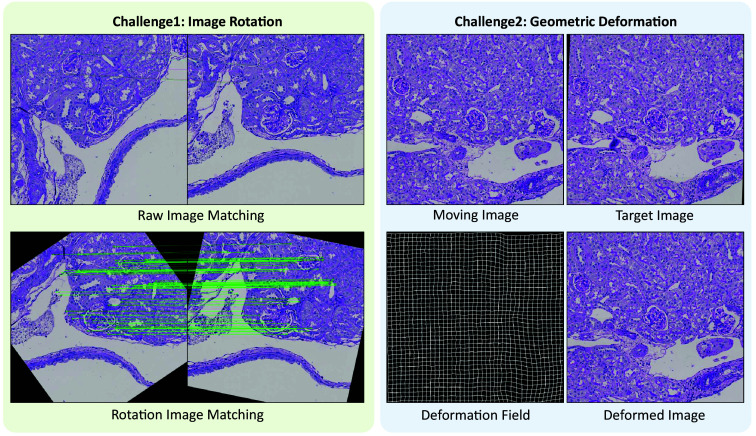
Challenges. This figure showed two major challenges in the registration process. The left panel showed the image rotation problem will affect the keypoint matching result. The right panel showed the geometric deformation between moving image and target image that need use non-rigid registration to solve help solving this problem.

Before performing the affine registration, we apply rotation preprocessing to the moving image to maximize the number of valid keypoint matching pairs with the fixed image. This preprocessing step enhances the registration accuracy by ensuring that the moving image is oriented optimally relative to the fixed image, thereby facilitating a higher number of accurate keypoint matches as shown in Fig. 3.

Rigid registration serves as the initial step in our registration pipeline, addressing global misalignments between consecutive histological sections. This stage ensures that the overall orientation, scale, and position of the tissue slices are consistent before finer adjustments are made. We employed XFeat^12^ as our image matching method for initial registration technique for the initial registration due to its speed and effectiveness.

### 3.2 Affine Registration

Building upon the robust keypoint matches obtained through our rotation invariant keypoint matching framework, we employ affine registration to achieve precise registration of consecutive histological sections. Affine registration models the transformation between two image slices as a combination of linear transformations (rotation, scaling, shearing) and translation, allowing for both global registration and local adjustments to accommodate minor deformations.

$$[\begin{array}{c} x^{\prime }\\ y^{\prime }\\ 1 \end{array}]=[\begin{array}{ccc} a_{11} & a_{12} & t_{x}\\ a_{21} & a_{22} & t_{y}\\ 0 & 0 & 1 \end{array}][\begin{array}{c} x\\ y\\ 1 \end{array}],$$ (1)

where (x,y) are the coordinates in the moving image $I_{t+1}$, $\left(x^{\prime },y^{\prime }\right)$ are the coordinates in the fixed image $I_{t}$, $a_{11}$, $a_{12}$, $a_{21}$, and $a_{22}$ are the elements of the affine transformation matrix A, and $t_{x}$ and $t_{y}$ are the components of the translation vector t.

To estimate the affine transformation parameters, we follow these steps as shown in Algorithm 1:

1. Keypoint detection and description: Keypoints are detected in both the fixed image $I_{t}$ and the moving image $I_{t+1}$ using XFeat^12^ after rotation preprocessing. Descriptors are computed for each keypoint to capture the local image structure.
2. Keypoint matching: The descriptors from the fixed and moving images are matched to establish correspondences between keypoints. This results in a set of matched pairs $M=\{(p_{i},q_{i})\}$, where $p_{i}$ is a keypoint in the fixed image and $q_{i}$ is the corresponding keypoint in the moving image.
3. Outlier rejection using RANSAC: To eliminate erroneous matches, we apply the RANdom SAmple Consensus (RANSAC) algorithm. RANSAC iteratively selects random subsets of matches, estimates the affine transformation, and computes the number of inliers that agree with this model. The transformation with the highest number of inliers is selected.
4. Affine transformation estimation: Using the set of inlier correspondences I⊂M, we estimate the affine transformation parameters by solving the following system of equations for each inlier pair $\left(p_{i},q_{i}\right)$.

**Algorithm 1:** Consecutive Affine Registration using Keypoint Matching and RANSAC.

**Require**: Image sequence $I_{1},I_{2},\ldots ,I_{N}$
1: **for** t=1 to N−1 **do**
2: **Set** Fixed image ← $I_{t}$
3: **Set** Moving image ← $I_{t+1}$
4: **Detect** keypoints and compute descriptors in Fixed and Moving images
5: **Match** keypoints between Fixed and Moving images to obtain set M
6: **Apply** RANSAC to M to filter out outliers, resulting in inlier set I
7: **Estimate** affine transformation parameters (A,t) using I
8: **Transform** Moving image using affine transformation: $x^{\prime }=\mathrm{Ax}+t$
9: **Optional:** Store transformation parameters or registered image
10: **end for**

### 3.3 B-spline Based Non-rigid Registration

Although rigid registration effectively addresses global misalignments, biological tissues often undergo local deformations that require more flexible transformation models. To capture and correct these local distortions, we employ B-spline based non-rigid registration, which allows for spatially varying transformations, enabling precise registration of complex anatomical structures by adapting to subtle morphological changes between sections as shown in Fig. 3.

The B-spline based non-rigid registration models the deformation field using a grid of control points overlaid on the image domain. The displacement of each pixel is calculated based on the displacements of the surrounding control points using B-spline basis functions. The transformation T(x) of a point x=(x,y) is given by

$$T(x)=x+\overset{n}{\underset{i=0}{\sum }}\overset{m}{\underset{j=0}{\sum }}c_{i,j}B_{i}(u)B_{j}(v),$$ (2)

where $c_{i,j}$ are the displacement vectors (control point coefficients) at control point (i,j). The functions $B_{i}(u)$ and $B_{j}(v)$ are the B-spline basis functions of degree k. The variables u and v are the normalized coordinates with respect to the control point grid. The parameters n and m denote the number of control points in the x and y directions, respectively.

The B-spline basis functions are defined recursively, providing smooth and continuous transformations suitable for modeling biological tissue deformations and the algorithm is shown in Algorithm 2.

#### 3.3.1 Loss function

To optimize the transformation parameters $\left\{c_{i,j}\right\}$, we used a combined loss function that incorporates both image similarity and transformation regularization

$$L=L_{\mathrm{NCC}}+\lambda L_{\mathrm{reg}},$$ (3)

where $L_{\mathrm{NCC}}$ is the local normalized cross-correlation (NCC) loss measuring the similarity between the fixed image $I_{t}$ and the transformed moving image $I_{t+1}$. The term $L_{\mathrm{reg}}$ is the regularization loss enforcing smoothness in the displacement field. The parameter λ is a weighting factor that balances the contribution of the regularization term.

The local NCC loss is defined as

$$L_{\mathrm{NCC}}=-\underset{x\in \Omega }{\sum }\frac{S_{\mathrm{num}}(x)}{\sqrt{S_{\mathrm{den}1}(x)}\sqrt{S_{\mathrm{den}2}(x)}},$$ (4)

where

$$S_{\mathrm{num}}(x)=\underset{r\in N(x)}{\sum }[I_{t}(r)-\mu_{t}(x)][I_{t+1}(T(r))-\mu_{t+1}(x)],$$ (5)

$$S_{\mathrm{den}1}(x)=\underset{r\in N(x)}{\sum }[I_{t}(r)-\mu_{t}(x){]}^{2},$$ (6)

$$S_{\mathrm{den}2}(x)=\sum_{r\in N(x)}[I_{t+1}(T(r))-\mu_{t+1}(x){]}^{2}.$$ (7)

In these equations, Ω is the image domain, and N(x) denotes a local neighborhood around point x. The terms $\mu_{t}(x)$ and $\mu_{t+1}(x)$ are the local mean intensities of the fixed and moving images within N(x), respectively.

The regularization loss $L_{\mathrm{reg}}$ is designed to encourage smoothness in the 2D displacement field u(x) by penalizing the squared differences of the displacement vectors among neighboring pixels, scaled relative to the image size. This diffusion regularization term is similar to VoxelMorph^28^ defined as

$$L_{\mathrm{reg}}=\frac{1}{2}(E_{x}[s_{x}^{2}\|\delta_{x}u(x){\|}^{2}]+E_{x}[s_{y}^{2}\|\delta_{y}u(x){\|}^{2}]),$$ (8)

where

$$\delta_{x}u(x)=u(x+1,y)-u(x,y),$$ (9)

$$\delta_{y}u(x)=u(x,y+1)-u(x,y).$$ (10)

In these equations, $u(x)=[u_{x}(x,y),u_{y}(x,y)]$ is the displacement vector at position x=(x,y). $\delta_{x}u(x)$ and $\delta_{y}u(x)$ are the finite differences of the displacement field along the x and y directions, respectively. $s_{x}=N_{x}$ and $s_{y}=N_{y}$ are scaling factors equal to the number of pixels in the x and y dimensions (i.e., the image size in each dimension). $E_{x}[\cdot ]$ denotes the expectation (mean) over all valid positions x in the image domain Ω. $\|\cdot {\|}^{2}$ denotes the squared Euclidean norm.

**Algorithm 2:** B-spline Based Non-Rigid Registration.

**Require:** Fixed image $I_{t}$, Moving image $I_{t+1}$, control point grid $\left\{c_{i,j}\right\}$, weighting factor λ, learning rate α, maximum iterations $N_{\max}$
1: Initialize control point displacements $c_{i,j}\leftarrow 0$ for all i,j
2: **for** iteration = 1 to $N_{\max}$ **do**
3: Compute the transformation T(x) using Eq. (2)
4: Warp the moving image: $I_{t+1}(T(x))$
5: Compute the loss L using Eqs. (3) and (4)
6: Compute gradients $\frac{\partial L}{\partial c_{i,j}}$ analytically or via automatic differentiation
7: Update control points: $c_{i,j}\leftarrow c_{i,j}-\alpha \frac{\partial L}{\partial c_{i,j}}$
8: **if** $|L^{\text{new}}-L^{\text{old}}|<\varepsilon$ **then**
9: **Break**
10: **end if**
11: **end for**
12: **Output:** Optimized transformation T(x)

## 4 Experiments

### 4.1 Data

#### 4.1.1 Mice datasets

In our study, we employed kidney tissue sections from both healthy and db/db diabetic mice to evaluate our computational models. Specifically, we analyzed 29 consecutive 2-μm-thick sections from normal mice and 39 consecutive 2-μm-thick sections from db/db diabetic mice. Each section was imaged at a high resolution of ×40 magnification to capture detailed histological features. The resulting images were subsequently divided into patches of size 2048×2048  pixels to facilitate scalable and efficient processing within our experiments.

#### 4.1.2 Human datasets

We have 20 human kidney biopsy cases, each containing 13 consecutive whole slide needle biopsy images. Each slide includes 2 to 5 tissue sections, with each section being 2  μm thick and captured at ×40 magnification. Each case undergoes sequential staining with three different stains—H&E first, followed by PAS and then Jones—repeated three times, with two unstained slides placed between each staining cycle. This results in the following sequence: H&E, PAS, and Jones, two unstained slides; H&E, PAS, and Jones, two unstained slides; and H&E, PAS, and Jones. To facilitate processing while maintaining detail, we downsample the whole slide images by a factor of two, making them more manageable without significant loss of information.

### 4.2 Evaluation Metrics

To quantitatively assess the performance of our registration method, we employ several evaluation metrics that measure the registration accuracy between the registered images and the ground truth landmarks.

Relative target registration error (rTRE), we followed the evaluation metric in Borovec’s work.^29^ The rTRE measures the Euclidean distance between corresponding landmarks after registration, normalized by the length of the image diagonal $d_{j}$. It provides a dimensionless metric to evaluate the accuracy of landmark registration.

rTREij,l=‖x^j,l−xj,l‖2dj, (11)

where

$$d_{j}=\sqrt{N_{x}^{j^{2}}+N_{y}^{j^{2}}},$$

is the diagonal length of image j, with $N_{x}^{j}$ and $N_{y}^{j}$ representing the dimensions of image j in the x and y directions, respectively. Here, x^j,l is the estimated position of landmark l in image j after registration, and $x_{j,l}$ is the ground truth position of the same landmark.

Average mean relative target registration error (AMrTRE) calculates the mean of the median rTRE values across all test image pairs. This metric provides an overall assessment of the registration accuracy by aggregating the central tendency of registration errors.

$$\mathrm{AMrTRE}=\frac{1}{\left|T\right|}\sum_{(i,j)\in T}\mu^{i,j},$$ (12)

where

$$\mu^{i,j}={\text{median}}_{l\in L^{i,j}}({\mathrm{rTRE}}_{ij,l}),$$

is the median rTRE for image pair (i,j). Here, T represents the set of all test image pairs, and $L^{i,j}$ is the set of landmarks present in both images i and j.

Median of median relative target registration error (MMrTRE) computes the median of the median rTRE values across all test image pairs. This metric offers a robust measure of registration accuracy by mitigating the influence of outliers

$$\mathrm{MMrTRE}={\text{median}}_{(i,j)\in T}(\mu^{i,j}).$$ (13)

Average maximum relative target registration error (AMxrTRE) calculates the mean of the maximum rTRE values for each test image pair. This metric highlights the average worst-case errors across the dataset, ensuring that extreme registration errors are accounted for

$$\mathrm{AMxrTRE}=\frac{1}{\left|T\right|}\underset{(i,j)\in T}{\sum }(\underset{l\in L^{i,j}}{\max}\;{\mathrm{rTRE}}_{ij,l}).$$ (14)

Average registration robustness ($R_{\mathrm{avg}}$) measures the proportion of landmarks for which the registration error decreased compared with the initial error before registration. This metric evaluates the consistency of the registration method in improving landmark registration.

$$R_{\mathrm{avg}}=\frac{1}{\left|T\right|}\underset{(i,j)\in T}{\sum }R^{i,j},$$ (15)

where

$$R^{i,j}=\frac{\left|K^{i,j}\right|}{\left|L^{i,j}\right|},$$

is the robustness for image pair (i,j) and

$$K^{i,j}=\{l\in L^{i,j}|{\mathrm{rTRE}}_{ij,l}<{\mathrm{rIRE}}_{ij,l}\},$$

is the set of successfully registered landmarks for image pair (i,j). Here, ${\mathrm{rIRE}}_{ij,l}$ is the relative initial registration error before registration, defined as

$${\mathrm{rIRE}}_{ij,l}=\frac{\|x_{i,l}-x_{j,l}{\|}_{2}}{d_{j}},$$

where $x_{i,l}$ is the initial position of landmark l in image i, assumed to be a reasonable approximation for $x_{j,l}$.

Average mean distance (AMean_D) measures the average Euclidean distance between corresponding landmarks after registration, without normalization. This metric provides an absolute measure of registration error

AMean_D=1|T|∑(i,j)∈T(1|Li,j|∑l∈Li,j‖x^j,l−xj,l‖2). (16)

### 4.3 Experiment Detail

All experiments were conducted on the same workstation with a 48-GB Nvidia RTX A6000. The registration image sequence was processed using 3D Slicer to generate 3D visualizations. The image spacing was set to 1  mm×1  mm×8  mm corresponding to a pixel size of 0.25  μm under ×40 magnification.

## 5 Results

**Fig. 4:**
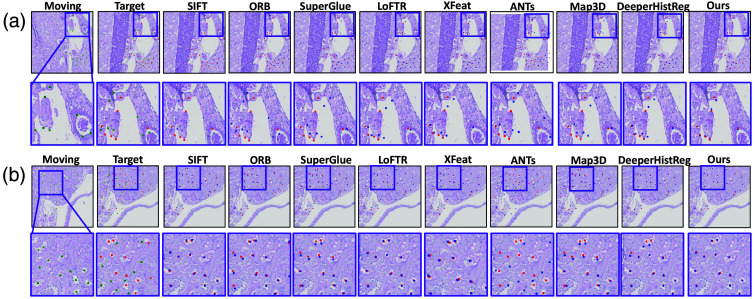
Qualitative result. This figure showed quantitative result between baseline method and our method. Part (a) shows the registration result on normal slice, and part (b) shows the registration result on db/db diabetic slice. Green points represent landmarks in the moving image, red points indicate landmarks in the reference image, and blue points denote the transformed landmarks after registration. The proximity between blue and red points reflects the registration quality, with closer registration indicating better performance.

Across the two mice datasets, our method consistently outperforms the baseline approaches in registration accuracy and robustness. As shown in Fig. 4, the qualitative results clearly demonstrate that our approach achieves lower error metrics across a variety of challenging scenarios compared to existing methods. This improvement is evident across different evaluation metrics, highlighting the adaptability and precision of our registration framework.

**Fig. 5:**
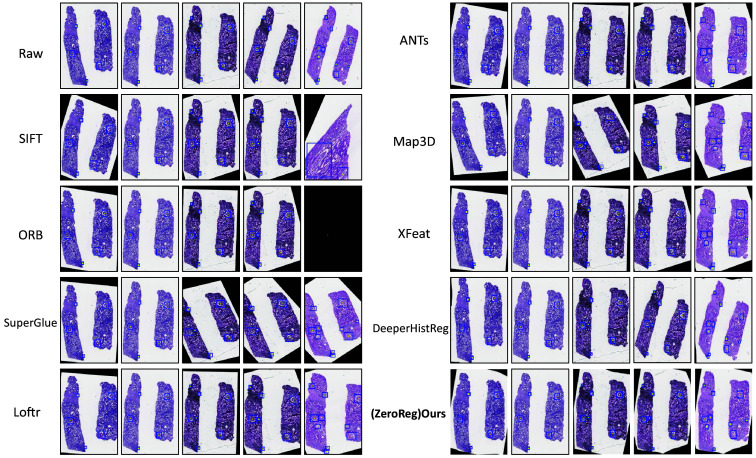
Qualitative result. This figure showed qualitative result between baseline method and our method on human whole slide images datasets. Each yellow point indicates the center points of the glomeruli, and the blue bounding box shows the size of the glomeruli.

Similarly, the evaluation on human datasets (see Fig. 5) reinforces the effectiveness of our method. The results indicate that our approach reliably produces lower AMean_D values across multiple test cases.

Specifically, in the first mice dataset, our method achieves the lowest AMrTRE (0.0024) and AMean_D (2.6952), indicating superior precision in aligning landmarks. In addition, it records a high R_avg (0.9872), demonstrating robust performance in consistently improving landmark registration as shown in Table 1.

**Table 1:** Comparison of registration methods on normal mice slice (unit μm).

Method	AMrTRE ↓	MMrTRE ↓	AMean_rTRE ↓	AMxrTRE ↓	R_avg ↑	AMean_D ↓
SuperGlue^13^	0.0263	0.0066	0.0275	0.0671	0.8738	19.9540
LoFTR^15^	0.0225	0.0048	0.0244	0.0527	0.8827	17.6954
XFeat^12^	0.0163	0.0049	0.0173	0.0383	0.9052	12.5473
SIFT^20^	0.0124	0.0106	0.0149	0.0395	0.7379	10.8311
ORB^21^	0.0211	0.0115	0.0227	0.0534	0.7008	16.4771
ANTs^25^	0.0527	0.0366	0.0532	0.0807	0.3214	38.5389
Map3D^26^	0.0268	0.0078	0.0278	0.0679	0.8392	20.1966
DeeperHistReg^27^	0.0026	0.0025	0.0043	0.0213	0.9726	3.1353
**ZeroReg3D (ours)**	**0.0024**	**0.0024**	**0.0037**	**0.0177**	**0.9872**	**2.6952**

In the second mouse dataset, our method maintains its leading performance with an AMrTRE of 0.0030 and an AMean_distance of 17.1674. Although some methods such as xfeat also demonstrate competitive AMrTRE (0.0160) and AMean_D (11.3080), our method still achieves the lowest values, underscoring its effectiveness. Notably, DeeperHistReg shows commendable performance with low AMrTRE (0.0064) and AMean_distance (4.9953), but our method surpasses it with even lower error metrics as shown in Table 2.

**Table 2:** Comparison of registration methods on db/db diabetic mice slice (unit μm).

Method	AMrTRE ↓	MMrTRE ↓	AMean_rTRE ↓	AMxrTRE ↓	R_avg ↑	AMean_D ↓
SuperGlue^13^	0.0160	0.0051	0.0160	0.0375	0.8537	11.6208
LoFTR^15^	0.0147	0.0036	0.0170	0.0465	0.9122	12.3183
XFeat^12^	0.0160	0.0038	0.0156	0.0374	0.8964	11.3080
SIFT^20^	0.0248	0.0086	0.0256	0.0548	0.5719	18.5785
ORB^21^	0.0262	0.0101	0.2708	0.0568	0.5965	19.6097
ANTs^25^	0.0438	0.0295	0.0441	0.0652	0.2882	31.9505
Map3D^26^	0.0249	0.0119	0.0247	0.0444	0.5265	17.8974
DeeperHistReg^27^	0.0064	**0.0025**	0.0068	**0.0218**	0.9464	4.9953
**ZeroReg3D (ours)**	**0.0030**	0.0027	**0.0059**	0.0273	**0.9754**	**4.2919**

**Fig. 6:**
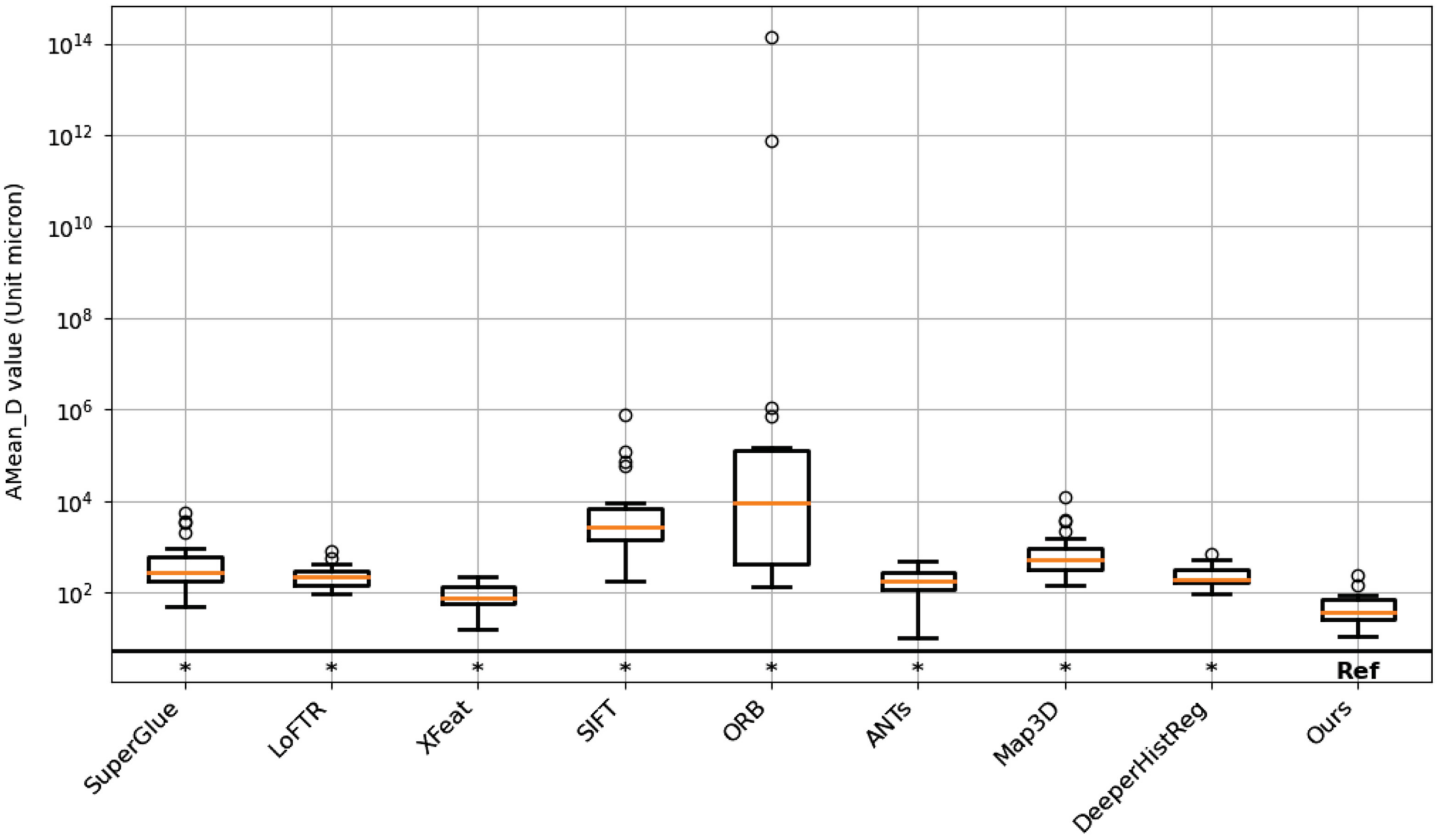
Statistical evaluation. This figure shows statistical significance evaluation between baseline methods and our method on human whole slide image datasets. The Wilcoxon signed-rank test (p<0.05) was used to assess statistical significance, with significant methods marked by an asterisk (*), indicating that our method consistently outperforms the other methods.

The human datasets shown in Fig. 6 compare the AMean_D values across nine methods over 20 human cases, with statistical significance assessed using the Wilcoxon signed-rank test. Our method exhibits the lowest median and least variability, indicating better and more stable performance than other methods. In contrast, SuperGlue, ORB, and SIFT show higher medians and larger spreads, suggesting greater variability and higher AMean_D values. Our method is significantly better than baseline methods on this dataset.

**Table 3:** Keypoints count before and after rotation preprocessing, along with timing statistics.

Dataset	Slice number	Keypoint pairs (before)	Keypoint pairs (after)	Time (s)	Time per slice (s)
Mice	66	12,965	16,882	5030	76
Human	505	289,529	323,029	142,380	281

Furthermore, several classical methods such as SIFT and ORB yield higher errors or exhibit missing values in multiple cases, underscoring their limitations in handling complex human datasets. In contrast, our method provides robust performance across all test cases and maintains competitive accuracy even in challenging scenarios where other advanced techniques falter.

We also evaluated the computational cost for the rotation preprocessing. For the mice dataset, which contains 66 slices, the number of keypoint pairs increased from 12,965 to 16,882 after applying the rotation step, with an average processing time of 76 s per slice. In the human dataset, consisting of 505 slices, the keypoint pairs increased from 289,529 to 323,029, with an average processing time of 281 s per slice.

### 5.1 Discussion

In this study, we introduced ZeroReg3D, a zero-shot registration pipeline designed to achieve precise and robust 3D reconstructions from serial histological sections. The superior performance observed across both mouse and human datasets can primarily be attributed to the careful integration of zero-shot deep learning-based keypoint extraction (XFeat) with robust affine and B-spline non-rigid registration methods. By combining deep learning techniques that inherently generalize well across diverse datasets with established mathematical models for deformation correction, our pipeline effectively mitigates common issues such as tissue deformation, sectioning artifacts, and variability in staining and illumination.

Specifically, the rotation preprocessing enhanced initial keypoint matching by ensuring optimal image orientation, thereby improving downstream affine and non-rigid transformations. The subsequent affine registration provided stable global registration, whereas the B-spline-based non-rigid registration captured subtle local tissue distortions, further refining the accuracy of registration. This holistic integration explains the consistent superiority of our method across various metrics, including lower AMrTRE, AMean_D, and higher average robustness scores compared to existing state-of-the-art methods.

In terms of computational cost on the mouse dataset shown in Table 3, ORB and SIFT required about 50 s and 730 s in total, respectively, whereas newer methods, LoFTR, a transformer-based matcher, and SuperGlue, a graph neural network-based matcher, took roughly 110 s and 40 s. XFeat, a lightweight feature extractor, further reduced total matching time to around 31 s. Complete registration frameworks such as ANTs and DeeperHistReg then add their optimization overhead, requiring ∼300  s and 260 s in total. Our complete pipeline uses XFeat matching in a single pass, completing in about 260 s, which is comparable to Map3D’s 400 s total. These results highlight that zero-shot matching with XFeat provides a favorable balance of accuracy and speed for mouse histology, making our approach especially practical for large-scale or resource-constrained studies.

Despite the strong performance, our method has limitations. One notable challenge is the computational cost associated with rotation preprocessing, which increases processing time. In addition, handling substantial discontinuities between histological sections, particularly observed in human datasets where the inclusion of unstained slides resulted in significant structural gaps, posed challenges. These gaps complicated keypoint correspondence and subsequent non-rigid registration, occasionally leading to suboptimal reconstructions. Another limitation arises from potential inaccuracies introduced during cumulative stacking of pairwise registration, especially in datasets with many serial sections, potentially propagating registration errors along the sequence.

Furthermore, compared with patch-level registration, WSI level registration presents greater challenges. For instance, the typical diameter of mouse glomeruli is ∼50  μm, with registration errors under 10  μm. In contrast, human glomeruli are larger—about 100  μm in diameter—but exhibit higher registration errors of about 50  μm. One possible explanation is that mouse datasets generally consist of 20 to 40 serial sections, whereas human datasets often contain around 30 to 60 serial sections, increasing the likelihood of cumulative registration errors. Another contributing factor is the presence of tissue drift, fragmentation, or missing regions in the serial WSIs, which further complicates the registration process.

Although our current study focuses on kidney tissue, it may be worthwhile to extend the proposed method to other tissue or organ types in the future studies. Future research will focus on addressing these limitations through several avenues. First, developing a rotation-invariant method could eliminate the need for rotation preprocessing, substantially reducing processing time. In addition, building an end-to-end registration pipeline could decrease the interpolation steps from two to one, further improving computational efficiency and potentially enhancing registration quality. Furthermore, we plan to explore global optimization methods that minimize cumulative registration errors over the entire sequence, potentially employing iterative refinement or joint registration techniques to enhance reconstruction fidelity.

## 6 Conclusion

In this study, we introduced ZeroReg3D, a zero-shot registration pipeline tailored for accurate 3D reconstruction from serial histological sections. By combining zero-shot deep learning-based keypoint matching with optimization-based affine and non-rigid registration techniques, ZeroReg3D effectively addresses critical challenges such as tissue deformation, sectioning artifacts, staining variability, and inconsistent illumination without requiring retraining or fine-tuning. Comprehensive evaluations demonstrated that our proposed pairwise 2D image registration method achieves ∼10% improvement in registration accuracy compared with state-of-the-art baseline methods, highlighting its superior precision and robustness. This framework provides a solid foundation for subsequent analysis transitioning from 2D serial sections to 3D histological tissue imaging.

## Data Availability

The code is available on GitHub. All datasets are in-house datasets and are not publicly available. The source code has been made publicly available at https://github.com/hrlblab/ZeroReg3D.
